# Desorption Techniques for Determination of Metals Mobility in Soils

**DOI:** 10.1100/tsw.2002.131

**Published:** 2002-03-05

**Authors:** P. Bartoš, F. Macášek

**Affiliations:** Department of Nuclear Chemistry, Faculty of Natural Sciences, Comenius University, Mlynska dolina CH-1, SK-84215 Bratislava, Slovakia

**Keywords:** metals, radiocaesium, mobility, soil, speciation, leaching

## Abstract

Three leaching techniques for assessment of fixed and mobile metal or radionuclides in soils are demonstrated on radiocaesium speciation. A new leaching technique based on the variation of the leaching solution volume to solid phase amount is proposed. It enables parallel treatment of large numbers of samples and, therefore, is suitable for a routine analysis of contaminant mobility in soils. As a leaching solution, 1 *M* ammonium acetate is proposed for caesium, but any other desorption solution harmonised with existing speciation schemes can be used.

## INTRODUCTION

From the view of soil contamination assessment, it is important to distinguish the fraction of contaminant in soil that is replaceable by desorption in real time from those fixed in silicate matrix during leaching, i.e., not entering exchange equilibrium and decreasing contaminant mobility in the environment[1]. During ageing of soil, the fraction of exchangeable ions changes continuously, and this limitation should be given some consideration if the method is to distinguish between permanently fixed and reversibly bound ions.

In the present work, three experimental techniques of caesium-mobility assessment based on ion desorption (leaching) of caesium from solid matrices are discussed.

## SRIP TECHNIQUE

The sorption capacity of minerals for caesium irreversibly trapped in collapsed micaceous layers at standard concentrations of potassium or ammonia was named *Specific Radiocaesium Intercept Potential* (SRIP)[2,3,4]. Determination of the SRIP value is based on the selective displacement of caesium from the weak "planar" sorption sites by the bulky molecules of a strong thiourea complex of silver ($Agtu_3^+$). From the distribution ratio, $D$(Cs) $dm^3\,kg^{-1}$, of caesium in 0.01 $M$ $KNO_3$–0.01 $M$ $Agtu_3NO_3$ solution, the SRIP value can be calculated[5] as

$$SRIP \approx D(Cs)\, c_K \text{ mol kg}^{-1} \quad (1)$$

where $c_K$ is potassium concentration in solution (in our case, $c_K = 0.01\ M$), or expressed through the soil activity (sample of mass *m)* before and after contact with volume $V$ of leaching solution, $A_0$ and $A$, respectively,

$$SRIP = c_K\, r\, A / (A_0 - A)\ [1 \pm 2\sqrt{}\ (\delta_0^2 + \delta^2)] \quad (2)$$

for a "two-sigma" level of confidence, where $\delta_0$ and $\delta$ are relative uncertainties of respective activities measurements, and $r$ is phase ratio (ml/g)

$$r = V/m \quad (3)$$

(usually $V/m$ = 100 ml/g, and therefore $c_K r = 1$ mol $kg^{-1}$).

However, the estimation of SRIP for many real soil samples[6] meets serious problems from the influence of humic substances and also high sensitivity of data to the equilibrium concentrations of caesium, which is of the order $10^{-6}$ $M$, a quite realistic figure in soil leachates. The presence of humic substances causes deposition of silver from the solutions, and SRIP is normally not applicable when the humic acids content in leachate exceeds 0.02 to 0.03 g/l[7].

## SEQUENTIAL LEACHING

The balance of gross activity, $A_o$, of metal in soil includes irreversibly sorbed (fixed, immobile) activity, $A_{fix}$, and the exchangeable (mobile) amount, $A_{ex}$,

$$A_0 = A_{fix} + A_{ex} \quad (4)$$

After leaching of activity, $A_{leach}$, the residual fraction of exchangeable activity is

$$E = (A_{ex} - A_{leach})/A_{ex} \quad (5)$$

and the balance changes to gross residual activity of soil

$$A = A_{fix} + A_{ex} - A_{leach} = A_{fix} + EA_{ex} \quad (6)$$

or, through a fraction of gross residual activity, $R = A/A_0$,

$$RA_0 = A_{fix} + EA_{ex} \quad (7)$$

In 1965, a sequential desorption technique was proposed for the purpose of distinguishing the reversible and irreversible parts of sorbed caesium[8] at which a set of residual activity fractions, $A_i$, after the $i$-th leaching step,

$$R_i = A_i/A_0\ (8)$$

is obtained and analysed. The distribution constant of caesium on the exchangeable sites can be expressed as

$$K_d = r\,(A_{ex} - A_{leach})/A_{leach} \quad (9)$$

or

$$K_d = r\,E/(1 - E) \quad (10)$$

where $r$ is the phase ratio, as calculated in Eq. 3. The fixed fraction value, $R_{fix} = A_{fix}/A_0$, and the residual fraction of soil activity in $i$ series of sequential leachings from Eqs. 7 and 8 is obtained as

$$R_i = R_{fix} + (1 - R_{fix})\,E^i \quad (11)$$

Then, $R_{fix}$ can be obtained from the first and second leaching data as follows:

$$(R_{fix})_{12} = (R_2 - R_1^2)/(1 + R_2 - 2R_1) \quad (12)$$

or from the 2nd and 4th leachings as

$$(R_{fix})_{24} = (R_4 - R_2^2)/(1 + R_4 - 2R_2) \quad (13)$$

Only when $R_1 \approx R_2$ is approved (i.e., the retaining activity remains nearly constant at two steps of leaching), $R_{fix} \approx R_1$, and a true fixed fraction value is found by single leaching. This is, however, not true for leaching caesium, e.g., from micas and bentonite, even with 1 *M* $KNO_3$ solutions and at a very favourable batch factor, $r$ = 50 ml/g[8].

Eq. 12 or 13 is used for algebraic calculations; otherwise iterative fitting should be applied to find both parameters $R_{fix}$ and $E$ in Eq. 11.

In our practice, 1 *M* ammonium acetate was chosen as a mild desorption agent with buffering properties for leaching 200 ± 5 mg of dried soil (sieved through 2-mm mesh screen) by 4 ml of solution for 2 h shaking. The technique gives good results (see Fig.1), but repeated operations of phase contact–centrifugation–aqueous phase removal–fresh leaching solution are time consuming and need hard-to-automate operations for a large number of samples.

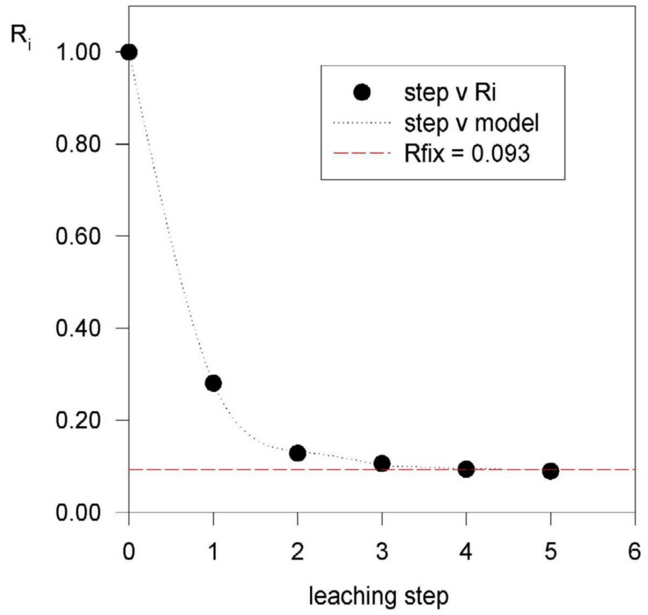

**FIGURE 1.** Sequential leaching of radiocaesium from montmorillonite by 1 *M* ammonium acetate (r = 100 ml/g, E = 0.21, and $K_d$ = 17).

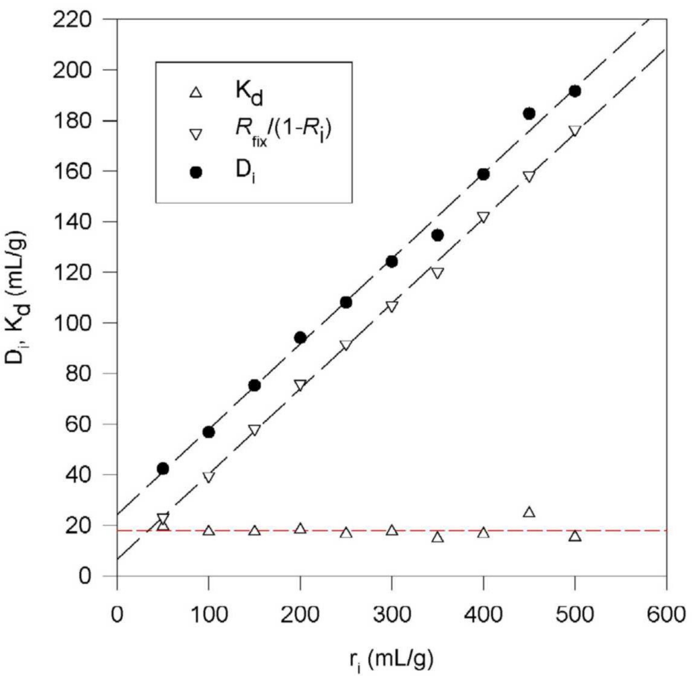

**FIGURE 2.** Radiocaesium leaching from montmorillonite by 1 *M* ammonium acetate at variable aqueous to solid phase ratios ($r_i$), $R_{fix}$ = 0.25, $K_d$ = 19 (for $r$ = 100, $E$ = 0.16)

Also, uncertainty of $R_{fix}$ from the $i$-th and $j$-th steps is proportional to $(1 - R_i)/(1 + R_j - 2R_i)^2$ and therefore strongly increases in vicinity of $R_j = 2R_i$[9].

## VARIABLE PHASE RATIO LEACHING

To achieve a time economy, a new leaching technique, which enables parallel treatment of large numbers of samples, is proposed.

Considering a single step of leaching, which is performed at various phase ratios, from Eqs. 10 and 11 it follows that the residual fraction of caesium is a function of variable phase ratio $r_i$:

$$R_i = R_{fix} + (1 - R_{fix})\frac{K_d}{r_i + K_d} \tag{14}$$

Eq. 10 can be solved for unknown $R_{fix}$ and $K_d$ (we used a nonlinear regression procedure of the SPSS Inc. SigmaPlot 4.01 program) and proved in linearized coordinates of gross distribution ratio of caesium, $D_i$ (ml/g), at the phase ratio $r_i$ (ml/g),

$$D_i = \frac{R_i}{1 - R_i} r_i = K_d + \frac{R_{fix}}{1 - R_i} r_i \tag{15}$$

Results obtained from 20-mg samples of contaminated montmorillonite K10 (Aldrich, 200 mesh) with 1 to 10 ml of 1 *M* ammonium acetate leaching solution after 2 h shaking and removal of aqueous phase by 10 min centrifugation at 4,000 rpm are presented in Fig. 2.

In principle, standard leaching solutions used for ions speciation in solids, 0.11 *M* acetic acid in particular[10], can be used instead of ammonium acetate, and samples (at least 250 mg) of soil powdered below 0.13 mm (about 100 mesh) can be recommended[11].

## ACKNOWLEDGEMENTS

Authors thank Slovak Grant Agency VEGA for financial support (Grant 1/7250/20).
